# Face First: The Role of Age and Sex in the Epidemiology of Facial Fractures

**DOI:** 10.3390/dj14010004

**Published:** 2025-12-22

**Authors:** Anna Aydin, Lawik Revend, Doha Revend, Manfred Giese, Oliver Schuck, Stephanie Roj, Johannes Schunk, Florian Dudde

**Affiliations:** 1 Department of Oral and Maxillofacial Surgery, Army Hospital Hamburg, Lesserstraße 180, 22049 Hamburg, Germany; 2 Department of Plastic Surgery, Army Hospital Berlin, 10115 Berlin, Germany; 3 Department of Otolaryngology-Head and Neck Surgery, Army Hospital Berlin, 10115 Berlin, Germany

**Keywords:** facial fractures, facial trauma, age, sex, risk factors

## Abstract

**Background:** Facial fractures are common in emergency and trauma care, with age and sex known to influence fracture patterns, injury mechanisms, and treatment approaches. However, detailed comparative data analyzing these demographic variables separately remain limited. **Methods:** In this retrospective single-center study, we analyzed 561 patients with radiologically confirmed facial fractures who were treated between January 2021 and December 2022. Patients were stratified by sex and age (<50 vs. ≥50 years). Fracture types, trauma causes, and treatment modalities were compared using odds ratios (ORs) with 95% confidence intervals (CIs). **Results:** Male patients comprised 60.1% of the cohort. Interpersonal violence, alcohol-related trauma, and sports injuries were significantly more frequent in males, while females experienced more falls and syncopes (*p* < 0.001). Although most fracture types did not differ significantly by sex, female patients underwent surgical treatment significantly less often than males (OR = 0.45, 95% CI: 0.32–0.64, *p* < 0.001). Patients over 50 years were significantly less likely to suffer mandibular fractures (OR = 0.59, 95% CI: 0.40–0.88, *p* = 0.009), while frontal sinus fractures were more common in older individuals (OR = 4.77, 95% CI: 1.02–22.27, *p* = 0.029). Younger patients more often experienced interpersonal violence, alcohol-related incidents, and received operative care, whereas falls and conservative treatment were more frequent among older individuals. **Conclusions:** Age and sex significantly influence the epidemiology and management of facial fractures. Understanding these demographic differences can guide targeted prevention strategies and assist clinical decision-making in facial trauma care.

## 1. Introduction

Facial fractures represent a significant portion of craniofacial trauma cases encountered in emergency care and maxillofacial surgical practice [1]. These injuries, ranging from mandibular and maxillary fractures to midfacial and orbital trauma, pose challenges not only in diagnosis and treatment but also in terms of long-term functional and esthetic outcomes [2,3]. The anatomical complexity of the facial skeleton, combined with the high-energy nature of many trauma mechanisms, makes facial fractures an important area of study in trauma epidemiology [3,4,5]. Multiple demographic and behavioral factors influence the incidence and pattern of facial fractures. Among these, age and sex have been consistently identified as relevant variables [6,7]. Studies have shown that younger patients tend to present with different trauma profiles and fracture distributions compared to older individuals [6,7,8]. Similarly, male and female patients often differ with regard to both the mechanisms of injury and the types of fractures sustained [6,7,8,9]. These distinctions have implications not only for trauma prevention but also for treatment planning and resource allocation in acute care settings. In the context of age, younger patients are more frequently involved in high-impact accidents such as sports-related injuries, road traffic collisions, and interpersonal violence [10]. These mechanisms are commonly associated with mandibular fractures and other injuries affecting the lower face [11]. In contrast, older individuals are more likely to experience falls or syncopes, which may result in other facial fractures [12]. Age-related changes in bone mineral density, balance, and reaction time are thought to contribute to this shift in injury pattern. In addition, older patients are often managed more conservatively due to comorbidities, reduced physiological reserves, or altered risk-benefit considerations with regard to surgical intervention [13,14].

Regarding sex, the existing literature has shown that male patients are generally at higher risk for facial trauma, particularly due to higher rates of alcohol use, physical altercations, and participation in high-risk activities [15,16]. In contrast, female patients more frequently sustain injuries resulting from accidental causes such as falls, and are more often treated non-operatively [17,18]. Some studies also suggest sex-based differences in the anatomical distribution of fractures and post-traumatic outcomes, although findings in this regard remain inconsistent [6,19]. While both age and sex have been recognized as important factors in facial trauma, few studies have evaluated these variables in sufficient detail using large, well-documented patient cohorts. To address this gap, we conducted a retrospective cohort study at a tertiary maxillofacial trauma center. The aim of this study was to analyze the influence of age and sex—separately—on the distribution, cause, and treatment of facial fractures. In doing so, we sought to provide a more nuanced understanding of the demographic determinants of facial trauma in a large patient population. By evaluating two separate comparative analyses—one based on age and one based on sex—we aimed to identify specific fracture patterns and treatment trends relevant to clinical practice and preventive public health strategies.

## 2. Materials and Methods

### Study Design and Participants

This retrospective, monocentric cohort study was conducted at the Department of Oral and Maxillofacial Surgery in a tertiary trauma and referral center. Patients treated between 1 January 2021 and 31 December 2022 were included. Eligible participants were of all ages and sexes and presented with at least one radiologically confirmed facial fracture. Patients were excluded if essential clinical documentation or demographic data were missing. All patients underwent standardized imaging following institutional trauma protocols. Computed tomography (CT) of the facial skeleton served as the primary diagnostic modality and was performed in all cases with clinically suspected facial fractures or moderate to high-energy trauma. In low-impact mechanisms or when an isolated mandibular injury was suspected, orthopantomography (OPG) was used as an adjunct or alternative modality. Only fractures that were radiologically confirmed, whether by CT or, in a minority of uncomplicated cases, by OPG, were included in the analysis. Because CT was the predominant modality, variability in imaging method is unlikely to have significantly influenced detection rates for the fracture subtypes reported. Patient data were extracted from the hospital’s electronic medical records and were identified via diagnostic codes and verified manually by two investigators to confirm fracture type and cause. All analyses were conducted on a per-patient basis. Each patient constituted a single observational unit, and fracture types were recorded as binary variables indicating the presence or absence of each subtype; multiple fractures in the same patient were not treated as independent observations. All radiologic and clinical records were reviewed independently by two investigators to confirm fracture type, trauma mechanism, and eligibility for inclusion. Discrepancies between reviewers were resolved through discussion and consensus. As this review step functioned primarily as a quality-control measure within a retrospective dataset, no formal inter-rater reliability statistics were calculated. The following variables were recorded: age, sex, type of fracture (e.g., mandibular, zygomatic, orbital floor, nasal bone, Le Fort classification, panfacial fractures), cause of injury (e.g., fall, sports accident, syncope, traffic accident, interpersonal violence), mode of treatment (surgical vs. non-surgical/conservative), and timing of the trauma event (weekday vs. weekend). Trauma circumstances were recorded using one primary etiological category per patient (fall, sports accident, syncope, road traffic accident, interpersonal violence). Additional contextual factors such as alcohol involvement, work-related injury, accident at home, or free-time accident were documented independently and were therefore non–mutually exclusive. Patients were stratified by age (<50 vs. ≥50 years) and by sex to allow for subgroup analyses. The age cutoff of 50 years was chosen because individuals above this threshold exhibit well-documented age-related physiological changes—such as decreased bone mineral density, impaired balance, and higher fall risk—which are known to alter both trauma mechanisms and fracture patterns. This cutoff is also consistent with previous epidemiological studies on facial trauma that stratify younger, high-impact injury populations from older, fall-dominated cohorts [6,7,8,10,11]. Primary outcomes were fracture type and treatment modality; secondary outcomes included trauma mechanism and temporal distribution. Statistical analyses were performed using SPSS (Version 28, IBM Corp., Armonk, NY, USA). Descriptive statistics were used to characterize the study population. Categorical variables were compared using chi-square tests; however, when expected cell frequencies were < 5 or zero—such as for rare fracture subtypes (e.g., Le Fort II and III)—Fisher’s exact test was applied. Non-categorical variables were compared using a *t*-test. Odds ratios (ORs) and 95% confidence intervals (CIs) were calculated to assess associations between age, sex, and fracture characteristics. Forest plots were generated for the visual representation of relevant associations. Associations were analyzed using unadjusted odds ratios because key potential confounders—such as fracture severity scores and comorbidity indices-were not consistently available in the retrospective dataset. Trauma mechanism, although recorded, demonstrated strong collinearity with age and sex, limiting the feasibility of meaningful multivariable adjustment. As such, the reported associations should be interpreted as descriptive rather than causal. A two-sided *p*-value < 0.05 was considered statistically significant.

## 3. Results

A total of 561 patients with radiologically confirmed facial fractures were included in the analysis. Of these, 337 (60.1%) were male and 224 (39.9%) were female (Table 1). The mean age was 53.19 ± 24.43 years (Table 1). The majority of fractures were observed in male patients and individuals under 50 years of age.

### 3.1. Analysis by Sex

**Figure 1:**
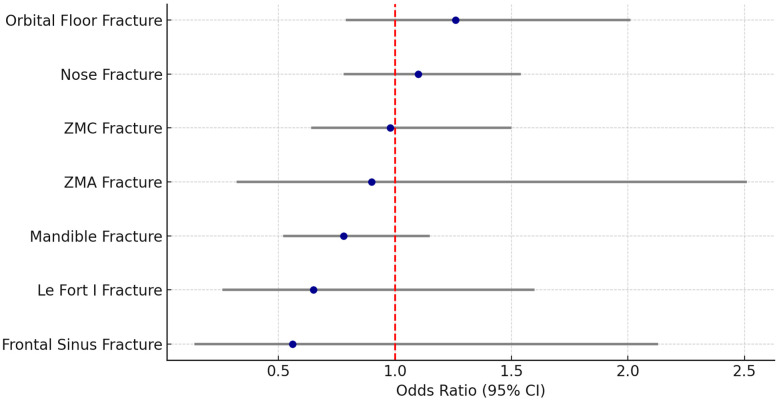
Forest plot facial fractures by sex. ZMC = Zygomatico-maxillary-complex, ZMA = Zygomatic arch, CI = confidence interval. Note: All non-significant.

Overall, only minor sex-based differences were observed in the distribution of specific facial fractures (Table 2, Figure 1). Mandibular fractures were slightly more common in male patients (27.0%) than in females (22.3%), but this difference was not statistically significant (OR = 0.78, 95% CI: 0.52–1.15, *p* = 0.211) (Table 2, Figure 1). Zygomaticomaxillary complex (ZMC) fractures were almost similar in frequency in males (19.6%) compared to females (19.2%) (OR = 0.98, 95% CI: 0.64–1.50, *p* = 0.909) (Table 2, Figure 1). Orbital floor fractures occurred more often in females than in males (females: 17.0% vs. Males: 13.9%, OR = 1.26, 95% CI: 0.79–2.01, *p* = 0.329) (Table 2, Figure 1). No significant sex-related differences were found for nasal fractures, ZMA fractures and panfacial trauma by sex. However, Le Fort I (non-significant) and Le Fort II fractures (significant) occurred more often in males (Table 2, Figure 1). A distinct sex-related trend was noted in treatment modalities: female patients underwent surgical treatment significantly less often than males (OR = 0.45, 95% CI: 0.32–0.64, *p* < 0.001), while conservative management was significantly more common among female patients (OR = 2.22, 95% CI: 1.55–3.17, *p* < 0.001) (Table 2).

Analysis of injury mechanisms marked sex-related differences in the circumstances of facial trauma (Table 3a,b). Falls were significantly more frequent among female patients (49.1%) compared to males (26.4%), with an odds ratio (OR) of 4.62 (95% CI: 3.21 –6.66, *p* < 0.001) (Table 3). Similarly, syncopes were more common in women (10.7% vs. 3.6%; OR = 2.17, 95% CI: 1.24–3.80, *p* = 0.006), whereas sports accidents occurred more frequently in men (15.7% vs. 4.5%; OR = 0.47, 95% CI: 0.29–0.77, *p* = 0.002) (Table 3).

Interpersonal violence was substantially less common in female patients (9.8% vs. 38.6%), with a highly significant odds ratio of 0.17 (95% CI: 0.11–0.28, *p* < 0.001) (Table 3). The same trend was observed for alcohol-related trauma, which was nearly three times more likely in males (28.2% vs. 11.6%; OR = 0.34, 95% CI: 0.21–0.54, *p* < 0.001), and work-related injuries (13.9% vs. 4.0%; OR = 0.35, 95% CI: 0.17–0.74, *p* < 0.001) (Table 3). Accidents at home were more frequently observed in female patients (35.3% vs. 29.7%; OR = 1.29, 95% CI: 1.12–1.95, *p* = 0.016), while free time accidents were also significantly more frequent in women (96.0% vs. 86.1%; OR = 3.87, 95% CI: 1.86–8.07, *p* < 0.001) (Table 3). No significant sex-based differences were observed regarding the day of injury: trauma occurrence on weekdays (males: 52.8%, females: 51.8%) and weekends (47.2% vs. 48.2%) was nearly identical (*p* = 0.810 for both comparisons) (Table 3).

### 3.2. Analysis by Age

**Figure 2:**
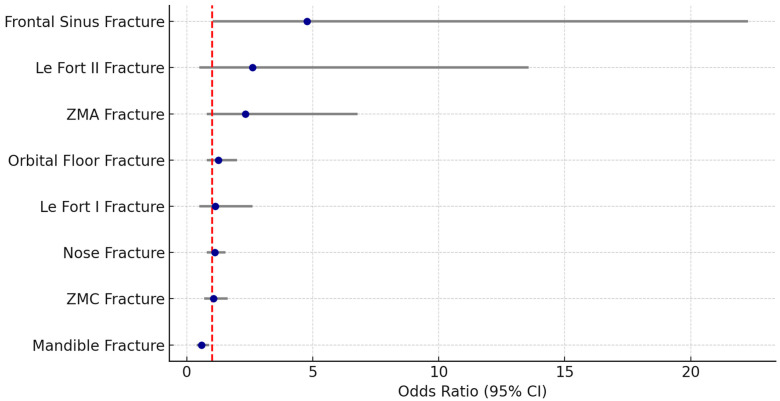
Forest plot facial fractures by Age. ZMC = Zygomatico-maxillary-complex, ZMA = Zygomatic arch, CI = confidence interval. Note: Significant differences for frontal sinus and mandible fractures.

A significant difference in sex distribution was also observed: the proportion of females was higher among the older individuals (57.2%) than in the younger group (23.2%) (*p* < 0.001) (Table 4). Mandibular fractures were significantly more common in younger patients (<50 years), occurring in 29.8% of cases compared to 20.3% among older individuals (≥50 years) (Table 5, Figure 2). This corresponds to an odds ratio (OR) of 0.59 (95% CI: 0.40–0.88, *p* = 0.009), indicating a significantly reduced likelihood of mandible fractures in the older age group (Table 5, Figure 2). In contrast, frontal sinus fractures occurred more frequently in older patients (2.2% vs. 0.2%), with a significant OR of 4.77 (95% CI: 1.02–22.27, *p* = 0.029) (Table 5, Figure 2). Although Le Fort II fractures were also more common in patients ≥ 50 years (2.2% vs. 0.7%), this difference did not reach statistical significance (OR = 2.61, 95% CI: 0.50–13.57, *p* = 0.254) (Table 5, Figure 2). No significant age-related differences were found for nasal, orbital floor, ZMC, ZMA, or Le Fort I fractures (Table 5, Figure 2). Regarding treatment, older patients were significantly less likely to undergo operative treatment (46.7% vs. 35.5%; OR = 0.63, 95% CI: 0.45–0.88, *p* = 0.007) (Table 5). Falls were significantly more frequent in patients aged 50 years and older (48.1%) compared to those under 50 years (23.2%), with an odds ratio (OR) of 9.95 (95% CI: 6.75–14.66, *p* < 0.001). Similarly, syncopes were more prevalent in the older group (10.1% vs. 2.8%; OR = 3.14, 95% CI: 1.69–5.82, *p* < 0.001) (Table 6a,b).

In contrast, interpersonal violence was significantly more common in younger patients (36.1% vs. 17.8%), yielding an OR of 0.04 (95% CI: 0.02–0.08, *p* < 0.001), and alcohol-related trauma was also predominantly observed in the younger group (37.2% vs. 5.4%; OR = 0.09, 95% CI: 0.05–0.17, *p* < 0.001) (Table 6a,b). Sports accidents were more likely to occur in patients under 50 (17.2% vs. 5.1%; OR = 0.38, 95% CI: 0.24–0.61, *p* < 0.001), while work-related accidents also occurred more often in the younger age group (13.7% vs. 6.2%; OR = 0.58, 95% CI: 0.22–0.88, *p* = 0.003) (Table 6).

Free time accidents were more frequent among older patients (93.8%) than in younger individuals (86.3%), corresponding to an OR of 2.42 (95% CI: 1.33–4.38, *p* = 0.003) (Table 6). The temporal distribution of injuries also differed: fractures in patients ≥ 50 years occurred more frequently on weekdays (59.4%) compared to the younger group (45.6%; OR = 1.75, 95% CI: 1.25–2.44, *p* = 0.001), whereas weekend trauma was significantly more common among younger patients (54.4% vs. 40.6%; OR = 0.57, 95% CI: 0.41–0.80, *p* = 0.001) (Table 6).

## 4. Discussion

This retrospective analysis of 561 patients with radiologically confirmed facial fractures aimed to investigate how age and sex influence fracture patterns, trauma mechanisms, and treatment decisions. The study identified several significant trends that not only confirm findings from previous literature but also provide new insights into the nuanced demographic profiles of patients sustaining facial trauma.

### 4.1. Sex-Based Differences

Consistent with prior studies, our data show that male patients represented the majority of facial trauma cases [20]. Although differences in specific fracture types between sexes were mostly non-significant, a notable trend emerged: males were more likely to sustain mandibular and Le Fort II fractures, while orbital floor fractures were slightly more common in females. Previous studies have similarly reported a higher incidence of mandibular fractures in men, often attributed to higher rates of alcohol-related violence and participation in riskier behaviors such as contact sports or motorized transport [6,15,16,21]. The predominance of orbital fractures in females may reflect lower-impact trauma mechanisms, such as falls or domestic accidents, which are less likely to affect the lower face and mandible [17,18].

Marked sex-specific differences were observed in the mechanisms of injury. Falls and syncopes accounted for a significantly higher proportion of facial trauma in female patients, whereas males were more commonly injured due to interpersonal violence, sports and alcohol-related accidents. These findings align with earlier epidemiologic research, which associates male sex with intentional trauma and alcohol abuse, while unintentional injuries like falls are more frequent among females, particularly in the elderly [16,17,22]. Notably, interpersonal violence was nearly four times more common in men than in women, underscoring the importance of targeted public health strategies for violence prevention and alcohol misuse, particularly in younger male populations.

A significant sex-related disparity was also found in treatment modality. Female patients were significantly less likely to undergo surgical management than their male counterparts. This may reflect both the lower severity of trauma in females and potential clinician bias or preferences in treatment strategy. Conservative approaches may be favored in older or more medically complex patients, of whom a larger proportion were female in this study. Similar trends have been documented in previous trauma research, suggesting the need for further investigation into the intersection of sex, injury severity, and therapeutic decision-making [13,14,23].

### 4.2. Age-Related Differences

Patients under 50 years of age were more likely to suffer mandibular fractures, interpersonal violence, alcohol-related trauma, sports and work accidents, and weekend injuries. These findings are consistent with the behavioral risk profiles of younger adults, who tend to be more physically active and socially mobile, thereby increasing their exposure to high-energy trauma mechanisms [10,11,24]. In contrast, patients aged ≥50 years were significantly more likely to sustain injuries due to falls and syncopes, both of which are strongly associated with aging-related physiological changes, including diminished balance, reduced bone density, and polypharmacy [12,13,25].

Fracture localization also varied by age. While mandible fractures were significantly more prevalent in younger patients, frontal sinus fractures occurred more frequently in older individuals. Although these injuries are relatively rare overall, the increased susceptibility of older patients to midfacial fractures may stem from structural weakening of the craniofacial skeleton with age [26,27]. Frontal bone involvement might also reflect less mobility during falls, where older individuals are unable to adequately protect their faces [12,28]. The relatively higher rate of Le Fort II fractures in the elderly, while not statistically significant, may reflect a similar mechanism.

Interestingly, younger patients were significantly more likely to receive operative treatment. This is not unexpected, as younger individuals are typically more resilient, with fewer comorbidities and a greater emphasis placed on restoring pre-injury function and esthetics [29]. In contrast, older patients were more frequently treated conservatively, which may reflect medical contraindications, patient preference, or the relative severity of trauma. Several studies support the notion that age-related treatment decisions are influenced not only by fracture complexity but also by systemic health status, highlighting the need for multidisciplinary care in elderly trauma populations [13,14,30].

### 4.3. Clinical Implications

Taken together, these findings reflect well-described behavioral, physiological, and risk-profile differences across demographic groups. Younger adults—especially males—are exposed to high-energy trauma due to violence, sports, and alcohol-related activities, directly explaining their higher rates of mandibular and complex fractures as well as operative interventions. In contrast, aging-related factors such as impaired balance, reduced bone density, frailty, multimorbidity, and polypharmacy increase the likelihood of low-impact falls and conservative management in older individuals. These mechanisms offer a coherent explanation for the demographic stratification observed in our cohort. The results of this study have several implications for clinical practice and healthcare policy. First, understanding the typical trauma profiles associated with different age groups and sexes can aid in the development of tailored prevention programs. For instance, public health campaigns addressing alcohol misuse and interpersonal violence may be particularly effective in reducing trauma incidence in younger men, while fall prevention and osteoporosis management may mitigate facial fractures in older adults. Second, age- and sex-specific treatment strategies could be refined to optimize outcomes. For example, more aggressive rehabilitation and surgical correction might be pursued in younger patients, while minimally invasive or conservative strategies could be prioritized in older, comorbid individuals.

Additionally, emergency departments and trauma centers should consider demographic trends when allocating resources. Awareness of peak trauma periods—such as weekends for younger patients—and common injury settings—such as home environments for older females—could guide staffing, diagnostic imaging protocols, and interdisciplinary consultation needs. Finally, these findings underscore the importance of incorporating demographic data into trauma registries and decision-support tools to enhance individualized patient care.

### 4.4. Limitations

Several limitations of this study warrant discussion. First, the retrospective, single-center design may limit generalizability. Although our institution is a tertiary referral hospital for maxillofacial trauma and treats a broad spectrum of cases, regional sociocultural factors and referral patterns may have influenced the observed epidemiological distribution of injuries. Second, although our cohort included more than 500 patients, the relatively small numbers in certain fracture subtypes (e.g., panfacial trauma, Le Fort III, frontal sinus fractures) reduced statistical power for subgroup analyses. Third, detailed clinical variables-such as standardized fracture severity scoring, soft-tissue injury grading, or indications for operative versus conservative management-were not consistently available in the retrospective dataset. These factors may interact with age and sex and could influence both fracture patterns and treatment decisions. Fourth, long-term outcomes, functional recovery, and complication or reoperation rates were not assessed, which limits conclusions regarding the prognostic implications of demographic differences. Fifth, comorbidities, medications, frailty markers, and socioeconomic status were not systematically recorded, although these variables likely contribute to trauma mechanism, fall risk, and therapeutic decision-making, particularly in older patients. In addition, imaging modality was not completely uniform: although CT was used for the majority of cases, isolated mandibular fractures were occasionally diagnosed based on OPG alone, which may introduce minor detection bias for specific fracture subtypes. Finally, analyses were based on unadjusted comparisons. Important confounders—including trauma mechanism, fracture severity, comorbidity burden, and lifestyle factors-were either unavailable or demonstrated substantial collinearity with age and sex, preventing robust multivariable modeling. Consequently, the associations reported in this study should be interpreted as descriptive epidemiological trends rather than causal relationships.

## 5. Conclusions

This study highlights the significant influence of age and sex on the epidemiology, mechanisms, and management of facial fractures. While younger male patients are predominantly affected by high-energy trauma such as interpersonal violence, sports injuries, and alcohol-related incidents, older individuals—particularly females—more frequently sustain fractures due to low-impact events such as falls and syncopes. Mandibular fractures were more common in younger patients, whereas frontal sinus injuries occurred more often in the elderly. Treatment strategies also differed: surgical intervention was more frequently employed in younger patients, while older individuals were more commonly treated conservatively. These findings underscore the need for age- and sex-specific prevention strategies and clinical decision-making in facial trauma care. Improved understanding of demographic risk profiles can inform resource allocation, enhance interdisciplinary planning, and ultimately contribute to more individualized and effective patient management.

## Figures and Tables

**Table 1:** Baseline Characteristics by Sex.

Variable	Total (*n* = 561)	Male (*n* = 337)	Female (*n* = 224)	*p*-Value
Age (years)	53.19 (±24.43)	45.55 (±21.51)	64.67 (±24.12)	**<0.001**
Gender				**<0.001**
Male	337 (60.1%)	337 (100%)	0 (0%)	
Female	224 (39.9%)	0 (0%)	224 (100%)	

Note: Data presented as mean (SD) and/or absolute values (percentage).

**Table 2:** Facial fractures by sex.

Variable	Total (*n* = 561)	Male (*n* = 337)	Female (*n* = 224)	OR	CI (95%)	*p*-Value
Mandible Fracture	141 (25.1%)	91 (27.0%)	50 (22.3%)	0.78	0.52–1.15	0.211
Nose Fracture	255 (45.5%)	150 (44.5%)	105 (46.9%)	1.10	0.78–1.54	0.582
ZMC Fracture	109 (19.4%)	66 (19.6%)	43 (19.2%)	0.98	0.64–1.50	0.909
Isolated Orbital Floor Fracture	85 (15.2%)	47 (13.9%)	38 (17.0%)	1.26	0.79–2.01	0.329
Isolated ZMA Fracture	16 (2.9%)	10 (3.0%)	6 (2.7%)	0.90	0.32–2.51	0.841
Le Fort I Fracture	23 (4.1%)	16 (4.7%)	7 (3.1%)	0.65	0.26–1.60	0.342
Le Fort II Fracture	7 (1.2%)	7 (2.1%)	0 (0%)	/ *	/ *	**0.030**
Le Fort III Fracture	3 (0.5%)	3 (0.9%)	0 (0%)	/ *	/ *	0.157
Frontal Sinus fracture	11 (2.0%)	8 (2.4%)	3 (1.3%)	0.56	0.14–2.13	0.387
Panfacial fracture	1 (0.2%)	1 (0.3%)	0 (0%)	/ *	/ *	0.414
Operation	231 (41.2%)	164 (48.7%)	67 (29.9%)	0.45	0.32–0.64	**<0.001**
Conservative Treatment	330 (58.8%)	173 (51.3%)	157 (70.1%)	2.22	1.55–3.17	**<0.001**

ZMC = Zygomatico-maxillary-complex, ZMA = Zygomatic arch. Note: Data presented as absolute values (percentage) and odds ratio (OR) with confidence interval (CI, 95%). * Due to the absence of events in one group, the odds ratio was 0 and the 95% confidence interval could not be calculated.

**Table 3:** (**a**) Primary etiologies (mutually exclusive) of facial fractures by sex. (**b**) Additional contextual factors (non-exclusive) by sex.

(**a**)						
**Variable**	**Total (*n* = 561)**	**Male (*n* = 337)**	**Female (*n* = 224)**	**OR**	**CI (95%)**	***p*-Value**
Fall	199 (35.5%)	89 (26.4%)	110 (49.1%)	4.62	3.21–6.66	**<0.001**
Sports accident	63 (11.2%)	53 (15.7%)	10 (4.5%)	0.47	0.29–0.77	**0.002**
Syncope	36 (6.4%)	12 (3.6%)	24 (10.7%)	2.17	1.24–3.80	**0.006**
Road Traffic accident (including car, motorcycle, bike, e-scooter)	111 (19.8%)	70 (20.8%)	41 (18.3%)	0.85	0.56–1.31	0.472
Interpersonal violence	152 (27.1%)	130 (38.6%)	22 (9.8%)	0.17	0.11–0.28	**<0.001**
(**b**)						
**Variable**	**Total (*n* = 561)**	**Male (*n* = 337)**	**Female (*n* = 224)**	**OR**	**CI (95%)**	***p*-Value**
Accident at home	179 (31.9%)	100 (29.7%)	79 (35.3%)	1.29	1.12–1.95	**0.016**
Alcohol related trauma	121 (21.6%)	95 (28.2%)	26 (11.6%)	0.34	0.21–0.54	**<0.001**
Work related accident	56 (10.0%)	47 (13.9%)	9 (4.0%)	0.35	0.17–0.74	**<0.001**
Free time accident	505 (90.0%)	290 (86.1%)	215 (96.0%)	3.87	1.86–8.07	**<0.001**
Weekday	294 (52.4%)	178 (52.8%)	116 (51.8%)	0.96	0.68–1.35	0.810
Weekend	267 (47.6%)	159 (47.2%)	108 (48.2%)	1.04	0.74–1.46	0.810

Note: Data presented as absolute values (percentage) and odds ratio (OR) with confidence interval (CI, 95%).

**Table 4:** Baseline characteristics by age.

Variable	Total (*n* = 561)	Age > 50 Years (*n* = 276)	Age < 50 Years (*n* = 285)	*p*-Value
Age (years)	53.19 (±24.43)	75.00 (±14.42)	32.06 (±8.11)	**<0.001**
Gender				**<0.001**
Male	337 (60.1%)	118 (48.2%)	219 (76.8%)	
Female	224 (39.9%)	158 (57.2%)	66 (23.2%)	

Note: Data presented as mean (SD) and/or absolute values (percentage).

**Table 5:** Facial fractures by age.

Variable	Total (*n* = 561)	Age < 50 Years (*n* = 285)	Age > 50 Years (*n* = 276)	OR	CI (95%)	*p*-Value
Mandible Fracture	141 (25.1%)	85 (29.8%)	56 (20.3%)	0.59	0.40–0.88	**0.009**
Nose Fracture	255 (45.5%)	126 (44.2%)	129 (46.7%)	1.11	0.79–1.54	0.548
ZMC Fracture	109 (19.4%)	54 (18.9%)	55 (19.9%)	1.06	0.70–1.62	0.769
Isolated Orbital Floor Fracture	85 (15.2%)	39 (13.7%)	46 (16.7%)	1.26	0.79–2.00	0.325
Isolated ZMA Fracture	16 (2.9%)	5 (1.8%)	11 (4.0%)	2.33	0.79–6.78	0.112
Le Fort I Fracture	23 (4.1%)	11 (3.9%)	12 (4.3%)	1.13	0.49–2.61	0.771
Le Fort II Fracture	7 (1.2%)	2 (0.7%)	5 (1.8%)	2.61	0.50–13.57	0.236
Le Fort III Fracture	3 (0.5%)	0 (0%)	3 (1.1%)	n.a. *	n.a. *	0.078
Frontal Sinus fracture	11 (2.0%)	2 (0.7%)	9 (3.3%)	4.77	1.02–22.27	**0.029**
Panfacial fracture	1 (0.2%)	0 (0%)	1 (0.4%)	n.a. *	n.a. *	0.309
Operation	231 (41.2%)	133 (46.7%)	98 (35.5%)	0.63	0.45–0.88	**0.007**
Conservative Treatment	330 (58.8%)	152 (53.3%)	178 (64.5%)	1.59	1.13–2.23	**0.007**

ZMC = Zygomatico-maxillary-complex, ZMA = Zygomatic arch. Note: Data presented as absolute values (percentage) and odds ratio (OR) with confidence interval (CI, 95%). * Due to the absence of events in one group, the odds ratio was 0 and the 95% confidence interval could not be calculated.

**Table 6:** (**a**) Primary etiologies (mutually exclusive) of facial fractures by age. (**b**) Additional contextual factors (non-exclusive) by age.

(**a**)						
**Variable**	**Total (*n* = 561)**	**Age < 50 Years (*n* = 285)**	**Age > 50 Years (*n* = 276)**	**OR**	**CI (95%)**	***p*-Value**
Fall	199 (35.5%)	66 (23.2%)	133 (48.1%)	9.95	6.75–14.66	**<0.001**
Sports accident	63 (11.2%)	49 (17.2%)	14 (5.1%)	0.38	0.24–0.61	**<0.001**
Syncope	36 (6.4%)	8 (2.8%)	28 (10.1%)	3.14	1.69–5.82	**<0.001**
Road Traffic accident (including car, motorcycle, bike, e-scooter)	111 (19.8%)	59 (20.7%)	52 (18.8%)	0.89	0.59–1.35	0.580
Interpersonal violence	152 (27.1%)	103 (36.1%)	49 (17.8%)	0.04	0.02–0.08	**<0.001**
(**b**)						
**Variable**	**Total (*n* = 561)**	**Age < 50 Years (*n* = 285)**	**Age > 50 Years (*n* = 276)**	**OR**	**CI (95%)**	***p*-Value**
Accident at home	179 (31.9%)	84 (29.5%)	95 (34.4%)	1.26	0.88–1.79	0.209
Alcohol related trauma	121 (21.6%)	106 (37.2%)	15 (5.4%)	0.09	0.05–0.17	**<0.001**
Work related accident	56 (10.0%)	39 (13.7%)	17 (6.2%)	0.58	0.22–0.88	**0.003**
Free time accident	505 (90.0%)	246 (86.3%)	259 (93.8%)	2.42	1.33–4.38	**0.003**
Weekday	294 (52.4%)	130 (45.6%)	164 (59.4%)	1.75	1.25–2.44	**0.001**
Weekend	267 (47.6%)	155 (54.4%)	112 (40.6%)	0.57	0.41–0.80	**0.001**

Note: Data presented as absolute values (percentage) and odds ratio (OR) with confidence interval (CI, 95%).

## Data Availability

The data presented in this study are not publicly available due to privacy restrictsions but can be obtained from the corresponding author upon reasonable request.
